# Assessment of genomic prediction reliability and optimization of experimental designs in multi-environment trials

**DOI:** 10.1007/s00122-021-03972-2

**Published:** 2021-11-22

**Authors:** Simon Rio, Deniz Akdemir, Tiago Carvalho, Julio Isidro y Sánchez

**Affiliations:** 1 Centro de Biotecnología y Genómica de Plantas (CBGP, UPM-INIA), Universidad Politécnica de Madrid (UPM) - Instituto Nacional de Investigación y Tecnologia Agraria y Alimentaria (INIA) Campus de Montegancedo-UPM, 28223 Pozuelo de Alarcón Madrid, Spain; 2 CIBMTR (Center for International Blood and Marrow Transplant Research), National Marrow Donor Program/Be The Match, Minneapolis, MN USA

## Abstract

**Key message:**

New forms of the coefficient of determination can help to forecast the accuracy of genomic prediction and optimize experimental designs in multi-environment trials with genotype-by-environment interactions.

**Abstract:**

In multi-environment trials, the relative performance of genotypes may vary depending on the environmental conditions, and this phenomenon is commonly referred to as genotype-by-environment interaction (G$\times$E). With genomic prediction, G$\times$E can be accounted for by modeling the genetic covariance between trials, even when the overall experimental design is highly unbalanced between trials, thanks to the genomic relationship between genotypes. In this study, we propose new forms of the coefficient of determination (CD, i.e., the expected model-based square correlation between a genetic value and its corresponding prediction) that can be used to forecast the genomic prediction reliability of genotypes, both for their trial-specific performance and their mean performance. As the expected prediction reliability based on these new CD criteria is generally a good approximation of the observed reliability, we demonstrate that they can be used to optimize multi-environment trials in the presence of G$\times$E. In addition, this reliability may be highly variable between genotypes, especially in unbalanced designs with complex pedigree relationships between genotypes. Therefore, it can be useful for breeders to assess it before selecting genotypes based on their predicted genetic values. Using a wheat population evaluated both for simulated and phenology traits, and two maize populations evaluated for grain yield, we illustrate this approach and confirm the value of our new CD criteria.

**Supplementary Information:**

The online version contains supplementary material available at 10.1007/s00122-021-03972-2.

## Introduction

In plant breeding, the best performing genotypes for a given trait are identified through field trials. Depending on the environmental conditions (e.g., temperature or rainfall), the relative performance of genotypes may vary, and this phenomenon is commonly referred to as genotype-by-environment interaction (G$\times$E, see Malosetti et al. (2013) and van Eeuwijk et al. (2016) for reviews). The characterization of G$\times$E involves the evaluation of genotypes in a set of environments representing the cultivation area: the so-called multi-environment trials (METs). If the G×E landscape is stable over the years, environments can be clustered into mega-environments that can be used to select genotypes with local adaptations (Gauch and Zobel 1997). Over the last decades, dedicated statistical methodologies have been developed to study G$\times$E including: (i) the regression proposed by Finlay and Wilkinson (1963), (ii) the additive main effect and multiplicative interaction (AMMI) model (Gauch and Zobel 1988; van Eeuwijk 1995), (iii) the genotype main effect and G$\times$E (GGE) model (Yan et al. 2000), (iv) the factorial regression modeling environmental covariates (Denis 1988; van Eeuwijk and Elgersma 1993), or (v) the random modeling of trial-specific genotype performances (Denis et al. 1997; Smith et al. 2005).

The advent of high-throughput genotyping has revolutionized the methods used to select for quantitative traits by enabling the genomic prediction of the genetic value of unobserved genotypes (Meuwissen et al. 2001). Regarding METs, genomic data can help to account for G$\times$E without the need to replicate genotypes within and across trials, as well as to predict the performance of genotypes in environments in which they have not been observed (Burgueño et al. 2012; Guo et al. 2013; Endelman et al. 2014; Ankamah-Yeboah et al. 2020; Jarquin et al. 2020). Several approaches have been proposed to account for G$\times$E in genomic prediction including adaptations of the GBLUP model based on linear (Burgueño et al. 2012; Jarquín et al. 2013; Lopez-Cruz et al. 2015; Crossa et al. 2016a) or non-linear kernels (Cuevas et al. 2016a; Cuevas et al. 2016b), approaches integrating crop growth models and genomic data (Heslot et al. 2013; Technow et al. 2015; Rincent et al. 2019), or deep learning methods (Montesinos-López et al. 2018).

In genomic selection, the ability to identify the best genotypes is closely related to the precision with which genetic values are predicted. This precision is most often evaluated using the correlation between the true and the predicted genetic values (then defined as the accuracy) or using the same correlation squared (then defined as reliability, here $R^2_G$). This precision can be assessed before the experiment using deterministic formulas based on population parameters (Daetwyler et al. 2008; Goddard et al. 2011) or other indicators like the coefficient of determination (CD) (VanRaden , 2008) which corresponds to the expected model-based $R^2_G$. In the context of GBLUP, CD depends only on variance parameters and the structure of the covariance (i.e., the genomic relationship matrix for genetic values and the identity matrix for errors). It has been used to forecast the $R^2_G$ using simulations (Goddard et al. 2011), animal (Hayes et al. 2009), or plant data (He et al. 2016). Adaptations of CD to complex covariance structures have been proposed and evaluated in the context of multi-group (Wientjes et al. 2015; Schopp et al. 2017; Rio et al. 2019) or multi-trait predictions (Ben-Sadoun et al. 2020). Such a statistical framework is also well suited to forecast $R^2_G$ in METs with G$\times$E, but to the best of our knowledge, it was not the subject of previous studies.

When designing METs, an important question is how to assign genotypes to plots to identify those that are best in each environment or for the overall cultivation area (i.e., regarding the mean performance). Several studies have focused on the allocation of genotypes to plots in one (de S. Bueno Filho and Gilmour , 2003; de S. Bueno Filho and Gilmour , 2007; Piepho and Williams , 2006; Butler et al. 2014) or more environments (Feoktistov et al. 2017; Rincent et al. 2017b; González-Barrios et al. 2019; Cullis et al. 2020) in presence of known kinship between genotypes. The ability of CD to forecast the $R^2_G$ makes it a valuable criterion to optimize experimental designs. A simple application is the selection of the genotypes to be included in a training set (TRS) among a large set of candidates using the mean of individual CDs (Rincent et al. 2012; Isidro et al. 2015; Rincent et al. 2017a; Akdemir and Isidro-Sánchez , 2019; Isidro-Sánchez and Akdemir , 2021). However, this scenario does not assume the existence of several environments for which genetic values may be different but correlated due to G$\times$E. In this context, an important question is whether accounting for this heterogeneity of genetic values between trials in the optimization procedure helps to obtain MET experimental designs that maximize the $R^2_G$ in presence of G$\times$E.

The objectives of this study were to (i) confirm the value of multivariate genomic prediction models in the context of METs with G$\times$E, (ii) present new CD criteria derived from those multivariate models and evaluate their ability to forecast the $R^2_G$ of both the trial-specific performance and the mean performance, (iii) evaluate the ability of new criteria to rank and optimize experimental designs based on the $R^2_G$. Note that the experimental designs tested in this study did not aim at evaluating the impact of the allocation of genotypes according to spatial characteristics within trials. Large publicly available wheat and maize datasets were used to address those objectives using both simulated and real traits.

## Material and methods

### Genomic prediction models

In this study, we considered a general multivariate model where response variables corresponded to the same trait in different trials. This multivariate model is presented here in a vectorized form:

1 $$\begin{aligned} \varvec{y=X\beta +Zg+e} \end{aligned}$$

where $$\varvec{y}=\begin{bmatrix}\varvec{y}_1\\ ...\\ \varvec{y}_T\end{bmatrix}$$ is the concatenated vector of phenotypes in *T* trials, $\varvec{\beta }=(\mu _1,...,\mu _T)^T$ is the vector of fixed trial means, $\varvec{X}$ is the design matrix for fixed effects, $$\varvec{g}=\begin{bmatrix}\varvec{g}_1\\ ...\\ \varvec{g}_T\end{bmatrix}$$ is the concatenated vector of random trial-specific genetic values with $\varvec{g}\sim \mathcal {N}(0,\varvec{G})$ and $\varvec{G}$ being the covariance matrix of $\varvec{g}$, $\varvec{Z}$ is the incidence matrix linking phenotypic observations to trial-specific genetic values, $\varvec{e}$ is the vector of errors with $\varvec{e}\sim \mathcal {N}(0,\varvec{R})$ and $\varvec{R}$ being the covariance matrix of $\varvec{e}$. Independence is assumed between $\varvec{g}$ and $\varvec{e}$.

Let us assume $\varvec{G}=\varvec{\Omega }_G\bigotimes \varvec{K}$ where $\varvec{K}$ is the genomic relationship matrix between genotypes (or any relationship matrix between them), and $\varvec{\Omega }_G$ is the genetic covariance matrix between trials. Similarly, let us assume $\varvec{R}=\varvec{\Omega }_E\bigotimes \varvec{I}_P$ where $\varvec{I}_P$ is the identity matrix of dimension *P* and $\varvec{\Omega }_E$ is the error covariance matrix between trials. Note that $\varvec{R}$ can be decomposed using a Kronecker product as the number of observations is the same for all trials.

Depending on the assumptions on $\varvec{\Omega }_G$ and $\varvec{\Omega }_E$, several genomic prediction models can be defined from Eq. (1):

- standard GBLUP (SGBLUP) with $$\varvec{\Omega }_G=\begin{bmatrix}\sigma _{G}^2 &{} .. &{}\sigma _{G}^2\\ .. &{} .. &{} .. \\ \sigma _{G}^2 &{} .. &{} \sigma _{G}^2\end{bmatrix}$$ and $$\varvec{\Omega }_E=\begin{bmatrix}\sigma _{E}^2 &{} .. &{}0\\ .. &{} .. &{} .. \\ 0 &{} .. &{} \sigma _{E}^2\end{bmatrix}$$ which amounts to applying a standard GBLUP model to the overall experimental design,
- within-trial GBLUP (WGBLUP) with $$\varvec{\Omega }_G=\begin{bmatrix}\sigma _{G_{1}}^2 &{} .. &{}0\\ .. &{} .. &{} .. \\ 0 &{} .. &{} \sigma _{G_{T}}^2\end{bmatrix}$$ and $$\varvec{\Omega }_E=\begin{bmatrix}\sigma _{E_{1}}^2 &{} .. &{}0\\ .. &{} .. &{} .. \\ 0 &{} .. &{} \sigma _{E_{T}}^2\end{bmatrix}$$ which amounts to applying a standard GBLUP model separately within each trial,
- multi-trial GBLUP (MGBLUP) with $$\varvec{\Omega }_G=\begin{bmatrix}\sigma _{G_{1}}^2 &{} .. &{}\sigma _{G_{1,T}}\\ .. &{} .. &{} .. \\ \sigma _{G_{T,1}} &{} .. &{} \sigma _{G_{T}}^2\end{bmatrix}$$ and $$\varvec{\Omega }_E=\begin{bmatrix}\sigma _{E_{1}}^2 &{} .. &{}0\\ .. &{} .. &{} .. \\ 0 &{} .. &{} \sigma _{E_{T}}^2\end{bmatrix}$$,

where $\sigma _{G}^2$ is the overall genetic variance, $\sigma _{G_{t}}^2$ is the genetic variance in trial *t*, $\sigma _{G_{t,t'}}$ is the genetic covariance between trials *t* and $t'$, $\sigma _{E}^2$ is the overall error variance and $\sigma _{E_{t}}^2$ is the error variance in trial *t*. Let us also define $\rho _{t,t'}=\dfrac{\sigma _{G_{t,t'}}}{\sigma _{G_{t}}\sigma _{G_{t'}}}$ as the genetic correlation between trials *t* and $t'$.

For all models, the estimation of parameters was done using a Bayesian framework. SGBLUP was implemented using the R package "BGLR" (Pérez and de los Campos , 2014) while WGBLUP and MGBLUP were implemented using the R package “MTM”^1^. Each inference consisted of 10,000 MCMC iterations with a burn-in of 1,000 iterations and a thinning of 5 (i.e., 1 out of 5 samples were conserved to compute posterior means). The prediction of each trial-specific genetic values was calculated as the sum of the estimated trial-specific mean ($\widehat{\mu }_t$) and the best linear unbiased predictor (BLUP) of $\varvec{g}_{it}$ ($\widehat{\varvec{g}}_{it}$). The BLUP of the complete vector $\varvec{g}$ can be computed as follows:

$$\begin{aligned} \widehat{\varvec{g}}=\varvec{GZ}^T\varvec{M}\varvec{y}, \end{aligned}$$

where $\varvec{M=\Sigma }^{-1}-\varvec{\Sigma }^{-1}\varvec{X}\left( \varvec{X}^T\varvec{\Sigma }^{-1}\varvec{X}\right) ^{-1}\varvec{X}^T\varvec{\Sigma }^{-1}$ and $\varvec{\Sigma }=\varvec{ZGZ}^T+\varvec{R}$ is the covariance matrix of $\varvec{y}$. The prediction of the mean genetic value over trials was computed as the mean of predicted trial-specific genetic values.

### Coefficient of determination

The CD (i.e., the expected model-based $R^2_G$) associated with the prediction of the genetic value of a genotype *i* in a trial *t* can be defined as:

2 $$\begin{aligned} {\begin{matrix} CD_{it}=&{}\mathrm {Cor}\left( \varvec{g}_{it},\widehat{\varvec{g}}_{it}\right) ^2\\ CD_{it}=&{} \dfrac{\left[ \varvec{GZ}^T\varvec{M}\varvec{ZG}\right] _{it,it}}{\varvec{G}_{it,it}}, \end{matrix}} \end{aligned}$$

Similarly, the CD associated with the prediction of the mean genetic value over all trials can be defined as:

3 $$\begin{aligned} {\begin{matrix} CD_{i.}=&{}\mathrm {Cor}\left( \frac{1}{T}\sum ^{T}_{t=1}\varvec{g}_{it},\frac{1}{T}\sum ^{T}_{t=1}\widehat{\varvec{g}}_{it}\right) ^2\\ CD_{i.}=&{}\dfrac{\sum ^{T}_{t=1}\sum^{T}_{t'=1}\left[ \varvec{GZ}^T\varvec{M}\varvec{ZG}\right] _{it,it'}}{\sum ^{T}_{t=1}\sum^{T}_{t'=1}\varvec{G}_{it,it'}} \end{matrix}} \end{aligned}$$

The derivation of $CD_{it}$ and $CD_{i.}$ may also be done based on Henderson’s mixed model equations (Henderson 1973) when the dimension of $\varvec{\Sigma }$ is larger than that of $\varvec{G}$, and/or using genetic correlations and variance ratios rather than the covariances matrices $\varvec{\Omega }_G$ and $\varvec{\Omega }_E$ (see Supplementary Note 1).

Three versions of $CD_{it}$ and $CD_{i.}$ were defined depending on the assumptions on $\varvec{\Omega }_G$ and $\varvec{\Omega }_E$ in the three genomic prediction models previously presented:

- Standard $CD_i^S$ associated to SGBLUP model for which $CD_i^S=CD^S_{it}=CD^S_{it'}=CD^S_{i.}$ for all *t*, $t'$,
- Within-trial $CD_{it}^W$ and $CD_{i.}^W$ associated to WGBLUP for which $CD_{i.}^W=\frac{1}{T}\sum ^T_{t=1}CD_{it}^W$,
- Multi-trial $CD_{it}^M$ and $CD_{i.}^M$ associated to MGBLUP for which $CD_{i.}^M>\frac{1}{T}\sum ^T_{t=1}CD_{it}^M$ when there exists a pair of trial for which $\rho _{t,t'}\ne 0$.

### Datasets

The first dataset considered in this study is a CIMMYT Iranian wheat population of 2,374 wheat inbred lines that have been genotyped for 40K SNPs (Crossa et al. 2016b; Montesinos-López et al. 2018; Montesinos-López et al. 2019). This population has been evaluated for days to heading (DTH) and days to maturity (DTM) in a drought trial and a heat trial at the CIMMYT experiment station (near Ciudad Obregon, Sonora, Mexico) in 2010–2011. Each trial was a grid-check field design with three randomized checks distributed along rows and columns (Crossa et al. 2016b).

The other datasets are two CIMMYT maize populations of 843 and 453 maize inbred lines that have been genotyped for 70K SNPs (Jarquin et al. 2020). The individuals of each population have been crossed to a different tester: T1 for the first population and T2 for the second population (T1 and T2 are further used to name each population). Both populations have been evaluated for grain yield in three trials (Drought, Kiboko, and Kmega) in Kenya, each being an incomplete block design.

In both datasets, each genotype has one reference phenotypic value after adjusting for spatial effects within trials. The genomic relationship matrices were computed following VanRaden (2008) and scaled so that the mean of diagonal elements is equal to 1. Note that, for both datasets, genotype-by-location and genotype-by-management interactions are probably confounded in the G$\times$E interaction. However, the various levels of genetic correlation between trials obtained for these real traits make them valuable data to illustrate the methodology presented in this study.

### Simulated traits

Using the wheat dataset only, traits were simulated for $T=2$ and $T=5$ trials based on the MGBLUP model and the set of parameters indicated in Table 1. Different levels of G$\times$E were simulated by adjusting the genetic correlation between trials: $\rho _{t,t'}\in \{0.2,0.5,0.8\}$. For a given simulated scenario (characterized by *T* and $\rho _{t,t'}$), the same $\rho _{t,t'}$ was considered between all pairs of trials. For each trial, genetic values were simulated as the sum between the trial-specific means $\mu _t$ and a deviation specific to each trial-specific genotype effect $\varvec{g}_{it}$. The vectors of correlated deviations were generated using the product between the Cholesky decomposition of $\varvec{G}$ (scaled with corresponding $\rho _{t,t'}$ and $\sigma ^2_{G_{t}}$ for all trials) and a vector of independent draws from a standard normal distribution. For each simulated scenario, 30 traits were simulated as replicates.

For each simulated MET experimental design, phenotypes were simulated by adding independent errors drawn from normal distributions with variance $\sigma ^2_{E_{t}}=\frac{1-h^2_t}{h^2_t}\sigma ^2_{G_{t}}$.

**Table 1:** Trial-specific parameters considered to simulate traits for $T=2$ and $T=5$ trials

Trial	1	2	3	4	5
Mean ($\mu _t$)	20	10	50	30	40
Genetic variance ($\sigma _{G_{t}}^2$)	1	2	3	4	5
Plot heritability ($h^2_t$)	0.2	0.8	0.6	0.4	0.5

Note that for $T=2$ trials, only the two first columns were used

### Comparison of genomic prediction models

Using the wheat dataset and simulated traits, the three genomic prediction models (SGBLUP, WGBLUP, and MGBLUP) were compared for their $R^2_G$. The cross-validation (CV) procedure consisted of defining a TRS of 500 genotypes observed once in each trial, with all other genotypes forming the test set (TS). For each of the 30 CV replicates, new phenotypes were generated (i.e., new errors were sampled to generate phenotypes). The $R^2_G$ was then computed as the square correlation between predicted and true genetic values for both the trial-specific and the mean performances.

### Link between the coefficient of determination and the genomic prediction reliability

Using the wheat dataset and simulated traits, the ability of $CD_{it}$ and $CD_{i.}$ criteria to forecast the $R^2_G$ was investigated based on the CV procedure presented in the previous subsection. To show how the CD associated with a genomic prediction is connected to the observed $R^2_G$, groups of predictions with homogeneous CD were defined according to the following procedure. For each CV sample and simulated trait, $CD_{it}$ and $CD_{i.}$ were computed for each genotype according to the design and the parameter estimates. Genotypes were then grouped according to sliding intervals of CD values: $[x-0.05,x+0.05]$ where $x\in \{0.05,0.10,...,0.90,0.95\}$. When two genotypes showed a kinship coefficient superior to 0.2 within an interval, one out of the two genotypes was discarded. This deletion procedure was applied iteratively until only "unrelated" genotypes remained within each interval. This procedure helped to limit the degree of correlation between the observations used to compute the observed $R^2_G$. Also, intervals containing less than 50 values were discarded from the analysis. For each interval, the corresponding $R^2_G$ was computed and compared to the reference value of the interval of *x*.

### Evaluation and optimization of experimental designs

Using the wheat dataset and simulated traits, all CD criteria were evaluated for their ability to forecast the $R^2_G$ that can be achieved by different experimental designs. To do so, the mean of each type of CD ($CD_{it}$ for the trial-specific performance and $CD_{i.}$ for the mean performance) was computed over the set of genotypes to be predicted and compared to the $R^2_G$ obtained for the same set using the MGBLUP model. The CV procedure to compare experimental designs consisted of separating the genotypes into a TS of 374 genotypes and a set of 2000 candidate genotypes from which the design of the experiment was defined.

**Fig. 1:**
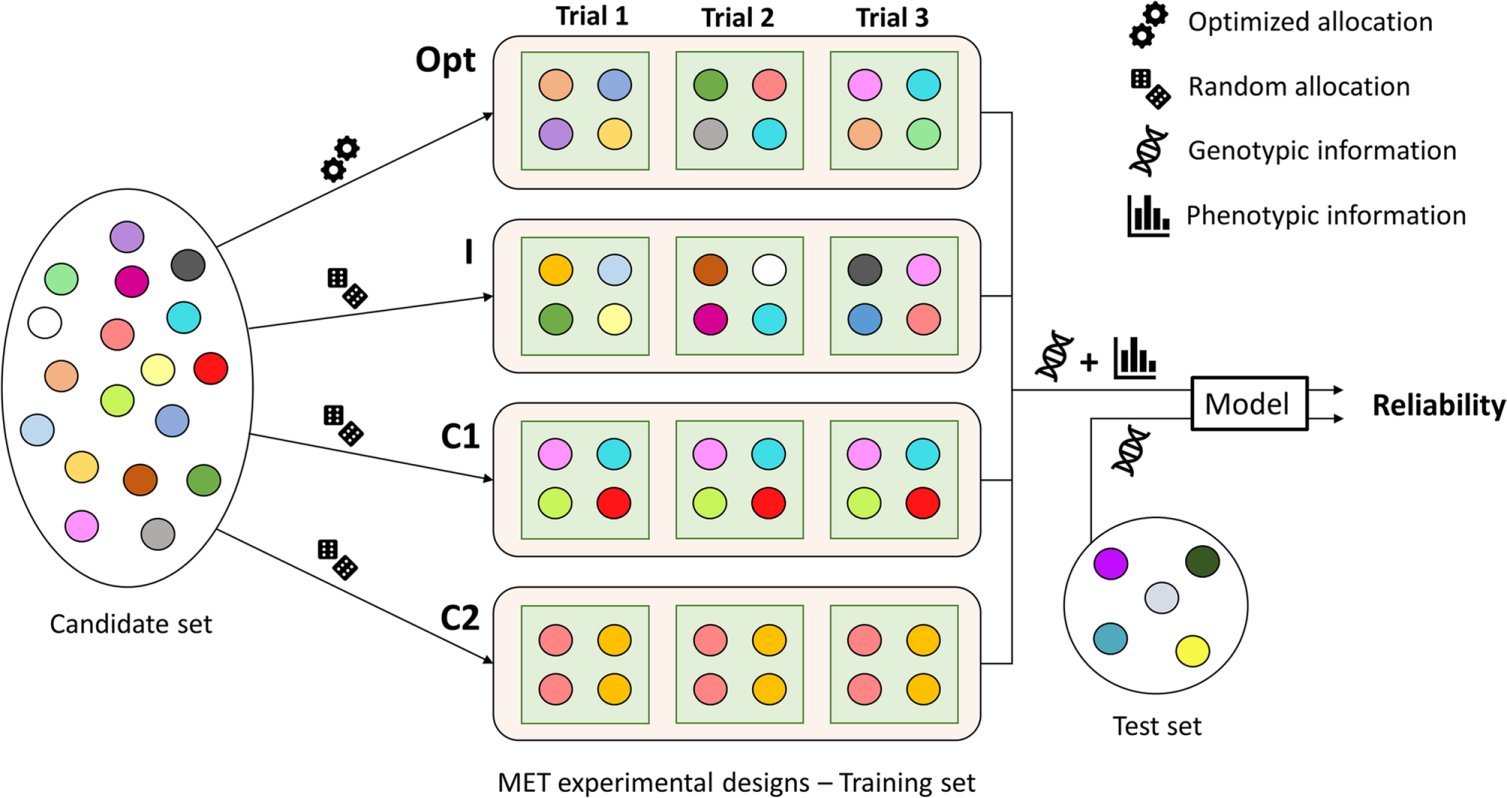
Diagram illustrating the procedure to allocate candidate set genotypes to MET experimental designs (Opt, I, C1, and C2) and predict the genetic value of test set individuals. Genotypes are allocated at random for designs I, C1, and C2, while they are allocated using an optimization approach based on CD criteria for the Opt design. Note that optimized designs do not allow for a genotype to be replicated within each trial, but they do allow a genotype to be replicated across trials

Different MET experimental designs were considered that are illustrated in Fig. 1:

- Incomplete and unreplicated design (I) where a given genotype is only observed once in a single trial,
- Complete design with *k* replicates (C*k*) where a given genotype is observed *k* times in each trial.

These MET experimental designs were compared to optimized experimental designs (Opt) obtained by maximizing either the mean of $CD_{it}$ over trials and genotypes, or the mean of $CD_{i.}$ over genotypes. These criteria were computed over the total set of candidates, excluding the genotypes of the TS. To limit the size of the set of possible solutions, experimental designs with replicates within trials were not considered in the optimization procedure. All CD criteria were computed for contrasted configurations of variance parameters $\varvec{\Omega }_G$ and $\varvec{\Omega }_E$ (Table 2) that matched the different genomic prediction models and CD types presented in previous subsections. The configuration S resumes to a standard univariate optimization based on the mean of $CD_i^S$. The configuration W corresponds to a multivariate optimization based on the mean of $CD_{it}^W$ (or equivalently $CD_{i.}^W$ as $CD_{i.}^W=\frac{1}{T}\sum ^T_{t=1}CD_{it}^W$). Note that the latter configuration resumes applying a standard univariate optimization based on $CD_i^S$ within each trial. The last configuration M corresponds to a multivariate optimization based on $CD_{it}^M$ or $CD_{i.}^M$ (not equivalent when there exists a pair of trials for which $\rho _{t,t'}\ne 0$).

**Table 2:** Trait parameters considered for the optimization according to the configuration: S, W or M. For a given configuration, the same variances ($\sigma ^2_{G_{t}}$ and $\sigma ^2_{E_{t}}$) were chosen for all trials, and the same genetic correlation ($\rho _{t,t'}$) was chosen for all pairs of trials

Configuration	S	W	M
Genetic variance ($\sigma ^2_{G_{t}}$)	1	1	1
Error variance ($\sigma ^2_{E_{t}}$)	1	1	1
Genetic correlation ($\rho _{t,t'}$)	1	0	0.5

The optimization procedure was implemented using the R-package "TrainSel"^2^ (Akdemir et al. 2021). The optimization algorithm implemented is a combination between a genetic algorithm (GA) and a simulated annealing algorithm (SAA). The algorithm is first initiated by a set of random designs. For each GA iteration, a population of 200 designs is generated from the elite set of designs obtained from the previous iteration. Such a step involves the recombination between elite designs and random mutations at the plot level. The best 20 designs are then selected according to the CD criterion and go through additional SAA iterations to limit the risk of finding a local optimum. A total of 2,000 iterations were considered for the GA and 10 iterations for the SAA at each iteration of the GA. Other parameters were default parameters. The convergence of the optimization algorithm was investigated by replicating the optimization procedure on the whole population 30 times.

### Application to real traits

Using the wheat and maize datasets and real traits, genomic prediction models and experimental designs were compared using the same CV procedure as for simulated traits, except that the same phenotypes were used for all CV replicates. For the comparison of models and experimental designs using the maize datasets, TSs of 138 and 53 genotypes were defined in populations T1 and T2. All remaining individuals were used as TRS for the comparison of models or as candidates for the comparison of experimental designs (50 plots per trial). The phenotype-based reliability of genomic prediction ($R_{Y}^2$) was computed as the square correlation between predicted genetic values and the reference phenotypic values.

### Data availability statement

The wheat dataset (genotypic and phenotypic data) is available at: http://hdl.handle.net/11529/10548141 (file: "Data_Wheat_Iranian_Set_3.RData.gz"). The maize datasets (genotypic and phenotypic data) are available at: http://hdl.handle.net/11529/10548369. Supplemental figures and tables are available in: "Supplementary file 1". R code is provided to apply the MGBLUP model, compute all CD criteria, and apply the optimization: "R_code.R" and "R_functions.R".

## Results

### Genomic prediction models comparison

**Fig. 2:**
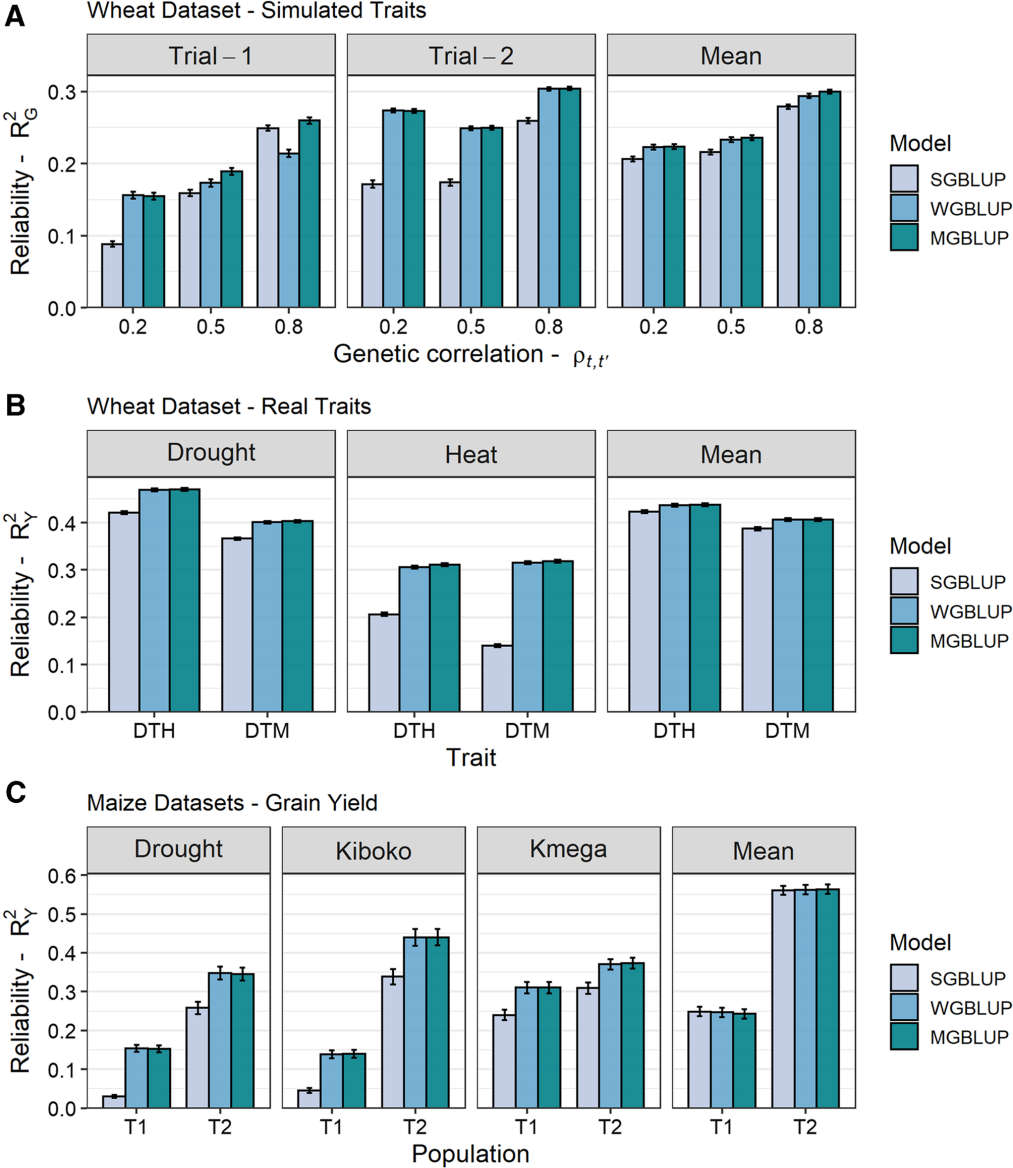
Comparison of the genomic prediction reliability ($R^2_G$ or phenotype-based $R^2_Y$) of different genomic prediction models (SGBLUP, WGBLUP, and MGBLUP) using maize and wheat datasets. For all datasets, the average $R^2_G$ (or $R^2_Y$) was illustrated for the trial-specific performance and the mean performance of the test set: **A** the wheat dataset with simulated traits (two trials: $h^2_1=0.2$ and $h^2_2=0.8$), **B** the wheat dataset with phenology traits (two trials: Drought and Heat), and **C** the maize dataset with grain yield (three trials: Drought and Kiboko and Kmega). For simulated traits, the average $R^2_G$ was computed over 30 simulated traits and 30 cross-validation replicates for each level of genetic correlation $\rho _{t,t'}$, and ± the standard error of the 30 CV replicates averaged over the 30 traits are indicated by a bar. For real traits, the average $R^2_Y$ is computed over 30 cross-validation replicates and ± the standard error is indicated by a bar

Based on the wheat dataset, the $R^2_G$ of three genomic prediction models (SGBLUP, WGBLUP, and MGBLUP) was evaluated using traits simulated under different scenarios defined by the number of trials and the levels of genetic correlation between trials (see Fig. 2A for $T=2$ and Supplementary Fig. S1 for $T=5$). The average $R^2_G$ of genomic prediction models were compared for both the trial-specific performance and the mean performance. For the mean performance, the average $R^2_G$ was very similar between models regardless of the genetic correlation between trials, even though a slight advantage from using MGBLUP could often be observed (e.g., for $\rho _{t,t'}=0.5$, the average $R^2_G$ was 0.22, 0.23 and 0.24 for SGBLUP, WGBLUP and MGBLUP, respectively). Regarding the trial-specific performance, the ranking of models was variable depending on the trial (each characterized by a specific heritability $h^2_t$), and the level of genetic correlation between trials. In general, SBGLUP achieved a lower average $R^2_G$ compared to other models, in particular for scenarios with a low genetic correlation between trials (e.g., for $\rho _{t,t'}=0.2$ in trial-2, the average $R^2_G$ was 0.17, 0.27 and 0.27 for SGBLUP, WGBLUP and MGBLUP, respectively). For most scenarios, WGBLUP achieved a similar average $R^2_G$ compared to MGBLUP. A notable exception consisted of trials with a low heritability and a high genetic correlation to other trials where both SGBLUP and MGBLUP outperformed WGBLUP (e.g., for $\rho _{t,t'}=0.8$ in trial-1 characterized by $h^2_1=0.2$, the average $R^2_G$ was 0.25, 0.21 and 0.26 for SGBLUP, WGBLUP and MGBLUP, respectively).

Using the wheat dataset and real phenology traits, the phenotype-based $R^2_Y$ of the same three models was compared (Fig. 2B). As for simulated traits, the average $R^2_Y$ for the mean performance was very similar between models for both traits. Regarding the trial-specific performance, the $R^2_Y$ was similar between MGBLUP and WGBLUP for all trials, while SGBLUP achieved a lower average $R^2_Y$ (e.g., for DTH in the drought trial, the average $R^2_Y$ was 0.42, 0.47, and 0.47 for SGBLUP, WGBLUP, and MGBLUP, respectively). The same observations could be made using the two maize datasets evaluated for grain yield (Fig. 2C).

### New CD criteria - simple example

**Table 3:** Comparison of simple experimental designs based on the new coefficient of determination (Coef. of Determination) criteria: $CD_{it}$ and $CD_{i.}$

	Ind.	Trial-1		Trial-2		Coef. of Determination		
						$CD_{i1}$	$CD_{i2}$	$CD_{i.}$
A	1	✓	✓	✓	✓	0.69	0.69	0.75
	2	–	–	–	–	0.00	0.00	0.00
	3	–	–	–	–	0.00	0.00	0.00
B	1	–	–	–	–	0.00	0.00	0.00
	2	✓	✓	✓	✓	0.69	0.69	0.75
	3	–	–	–	–	0.17	0.17	0.19
C	1	✓	–	✓	✓	0.54	0.68	0.69
	2	–	✓	–	–	0.50	0.12	0.33
	3	–	–	–	–	0.13	0.03	0.08
D	1	✓	–	✓	–	0.53	0.53	0.60
	2	–	✓	–	–	0.51	0.22	0.39
	3	–	–	–	✓	0.22	0.51	0.46

Each experimental design consisted of $T=2$ trials of $P=2$ plots each, with observed genotypes indicated by a cross (✓). The following parameters were considered: $$\varvec{K}=\begin{bmatrix}1&{}0&{}0\\ 0&{}1&{}0.5\\ 0&{}0.5&{}1\end{bmatrix}$$, $$\varvec{\Omega }_G=\begin{bmatrix}1&{}0.7\\ 0.7&{}2\end{bmatrix}$$ and $$\varvec{\Omega }_E=\begin{bmatrix}1&{}0\\ 0&{}2\end{bmatrix}$$, which resumes to consider a genetic correlation between trials $\rho _{1,2}\approx 0.5$ and a trial-specific heritability of $h^2_{t}=0.5$ for both trials. For this example, fixed effects were assumed to be known which resumes to replacing the $\varvec{M}$ matrix by $\varvec{\Sigma }^{-1}$ in Eq. (2, 3)

The properties of the new CD criteria ($CD_{it}^M$ and $CD_{i.}^M$) were illustrated based on a simple example (Table 3). Different experimental designs with two trials ($T=2$), two plots per trial ($P=2$), and three genotypes (only genotypes 2 and 3 are related to each other) were considered. For design A, only individual 1 is observed in both trials, leading to a high CD associated with both the trial-specific performances ($CD_{1t}$) and the mean performance ($CD_{1.}$). Conversely, null $CD_{it}$ and $CD_{i.}$ are obtained for genotypes 2 and 3, as none of them are observed in any trials and none of them are related to genotype 1. A higher $CD_{1.}$ compared to the mean of $CD_{1t}$ results here from the positive covariance between trials. For design B, only genotype 2 is observed in both trials, resulting in high $CD_{it}$ and $CD_{i.}$ for genotype 2 but null for genotype 1. As genotype 3 is related to genotype 2, both $CD_{3t}$ and $CD_{3.}$ takes small but non-null values. For design C, genotypes 1 and 2 are observed in trial-1, while only genotype 1 is observed in trial-2. As a result, genotype 2 has a high $CD_{21}$ but a low $CD_{22}$. Because genotype 3 is related to genotype 2, $CD_{31}$ is small but non-null, and because of the positive covariance between trials, $CD_{32}$ is very small but also non-null. The last design D is one of the possible optimal designs as it allows for the highest average $CD_{it}$ or $CD_{i.}$ over the three genotypes. The higher $CD_{i.}$ obtained for genotype 3 compared to genotype 2 results from the larger genetic variance in trial-2 compared to trial-1. Note that another optimal design would consist of switching genotypes 2 and 3 between trial-1 and trial-2.

### Expected and observed genomic prediction reliability

**Fig. 3:**
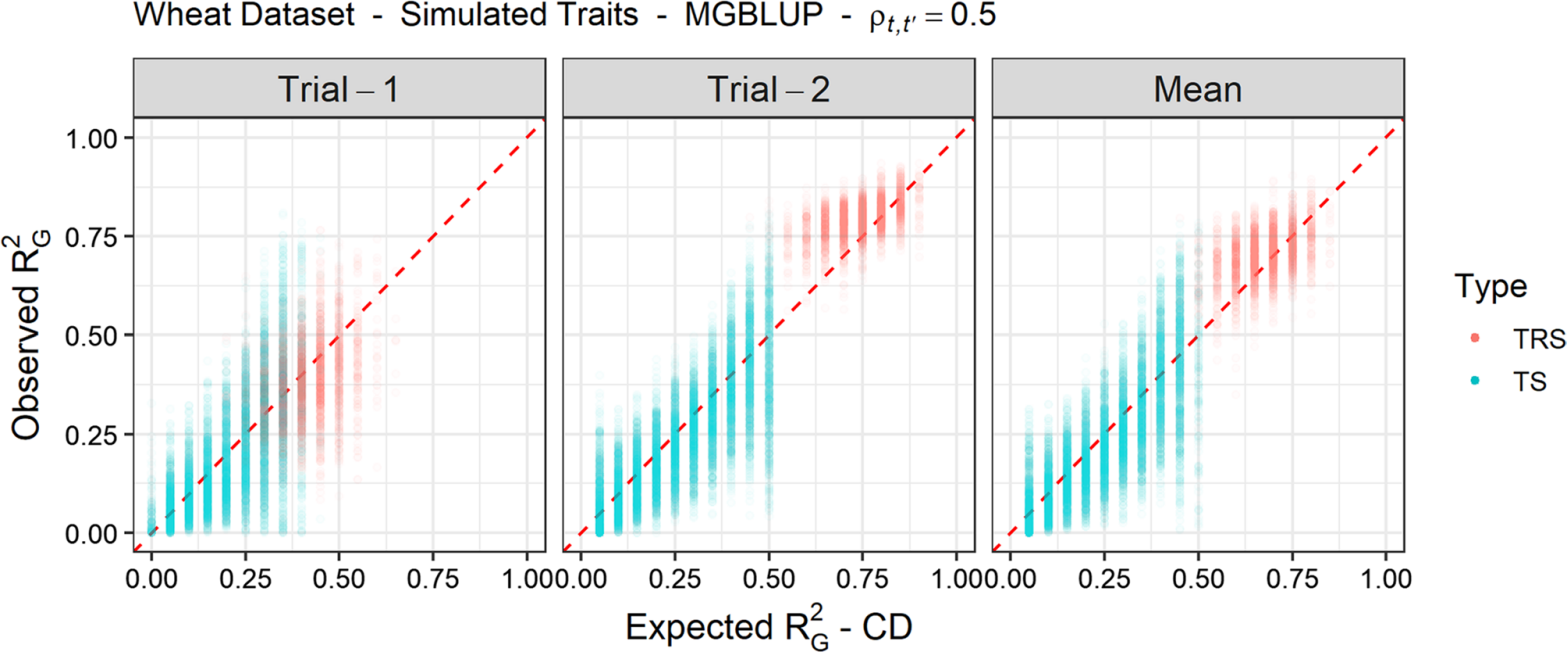
Observed genomic prediction reliability ($R^2_G$) against expected $R^2_G$ for 30 simulated traits ($\rho _{t,t'}=0.5$ and $T=2$ with different $h^2_t$, see Table 1) and 30 cross-validation replicates for the training set (TRS) and the test set (TS). Expected $R^2_G$ is based on $CD_{it}$ for trial-specific performances and $CD_{i.}$ for the mean performance. Individual CD values are clustered into groups of values with similar CD using a sliding window approach and the observed $R^2_G$ of the interval is then computed based on genomic predictions obtained with MGBLUP

The ability of the new CD criteria to forecast the $R^2_G$ of MGBLUP was investigated based on the CV replicates obtained for simulated traits with $\rho _{t,t'}=0.5$ (see Fig. 3 for $T=2$ and Supplementary Fig. S2 for $T=5$). The expected $R^2_G$ based on $CD_{it}$ and $CD_{i.}$ was generally a good indication of the level of observed $R^2_G$ for both the TRS and the TS. A substantial variability could be observed between the expected and observed $R^2_G$, especially for the TS.

For real traits, the expected $R^2_G$ was compared to the observed phenotype-based $R^2_Y$ jointly for DTH and DTM (see Fig. S3). Those quantities are not expected to be equal, but a clear trend could be observed in that the expected $R^2_G$ was correlated to the observed $R^2_Y$.

### Comparison and optimization of experimental designs

**Fig. 4:**
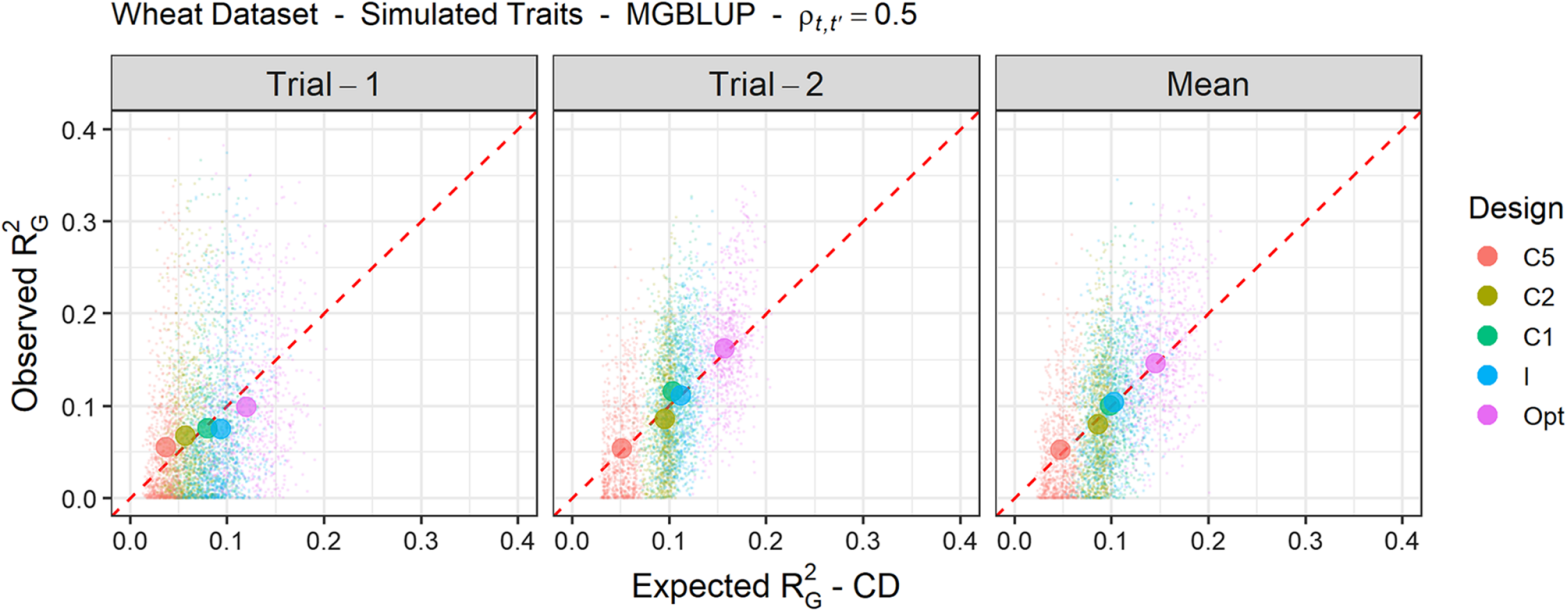
Observed genomic prediction reliability ($R^2_G$) obtained with MGBLUP against expected $R^2_G$ of the test set according to the experimental design: complete with *k* replicates (C*k*), incomplete and unreplicated (I), and optimized based on $CD_{it}$ with the M configuration (Opt, see Table 2), for 30 simulated traits ($\rho _{t,t'}=0.5$ and $T=2$, with different $h^2_t$, see Table 1) and 30 design replicates. Expected $R^2_G$ is based on the mean over test set individuals of $CD_{it}$ for the trial-specific performances and $CD_{i.}$ for the mean performance. For a given type of design, the average observed $R^2_G$ against the average expected $R^2_G$ is represented by a big dot

The ability of the new CD criteria to forecast the $R^2_G$ achieved by standard experimental designs was investigated using MGBLUP and simulated traits (see Fig. 4 for $T=2$ and Supplementary Fig. S4 for $T=5$ using $\rho _{t,t'}=0.5$). The $R^2_G$ achieved by complete designs with replicates (C*k*) were in general lower with an increasing number of replicates. The $R^2_G$ achieved by the incomplete design (I) was either comparable to that of C1 for $T=2$ or higher than that of C1 for $T=5$. On average, the expected $R^2_G$ based on the mean of $CD_{it}$ or $CD_{i.}$ was a good indication of the level of observed $R^2_G$.

Those standard experimental designs were compared to optimized experimental designs obtained based on $CD_{it}$ or $CD_{i.}$ with the contrasted parameter configurations S, W and M presented in Table 2. The algorithm did not converge as optimized designs were not identical between different initializations (Supplementary Table S1). However, each criterion clearly reached a plateau indicating very similar expected $R^2_G$ between design replicates (Supplementary Fig S5). The optimization configuration had little impact on the observed $R^2_G$ of experimental designs for simulated traits regardless of the simulated genetic correlation between trials, although designs optimized with configuration S generally achieved the lowest $R^2_G$ (see Supplementary Table S2). For the configuration M, using the mean of $CD^M_{i.}$ as a criterion is different from using the mean of $CD^M_{it}$, but did not allow for any improvement. Optimized designs based on $CD_{it}$ with configuration M were compared to standard experimental designs and showed an average improvement in expected $R^2_G$ that translated into an average gain in observed $R^2_G$ (for $\rho _{t,t'}=0.5$, see Fig. 4 for $T=2$ and Supplementary Fig. S4 for $T=5$).

**Fig. 5:**
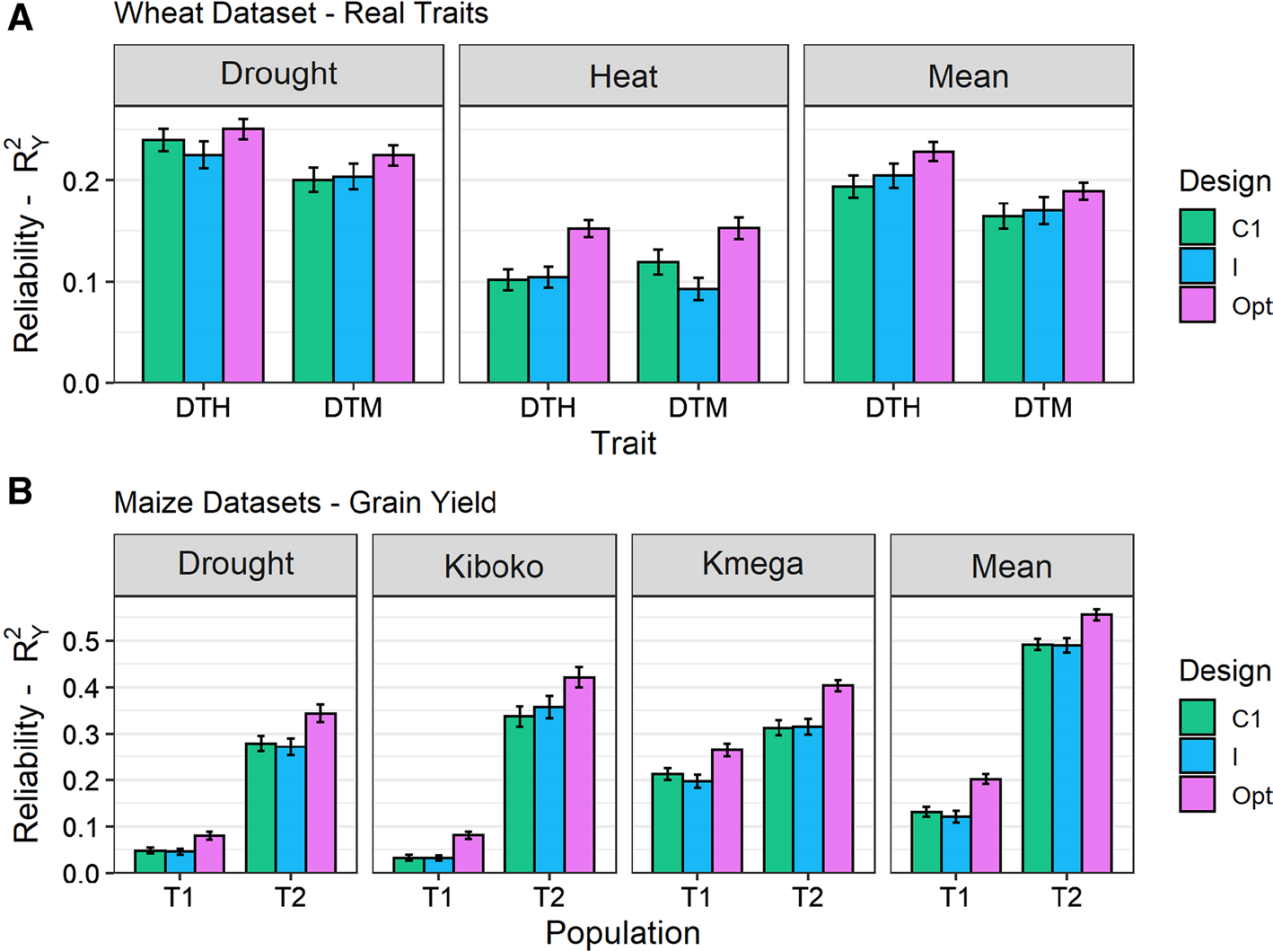
Average phenotype-based genomic prediction reliability ($R^2_Y$) of **a** days-to-heading (DTH) and days-to-maturity (DTM) for the wheat dataset and **b** grain yield for the two maize populations (T1 and T2), obtained with MGBLUP of the trial-specific and the mean performance of the test set according to the experimental design: complete with one replicate (C1), incomplete and unreplicated (I) and optimized based on $CD_{it}$ with the M configuration (Opt, see Table 2). The average $R^2_G$ was computed over 30 design replicates and ± the standard error is indicated by a bar

For real traits in all datasets, optimized experimental designs were obtained based on $CD_{it}$ with the configuration M generally allowed for higher phenotype-based $R^2_Y$ compared to standard designs, both for the trial-specific performance and the mean performance (Fig. 5). Like for simulated traits, the impact of the optimization configuration was limited for both traits, except for designs optimized with configuration S, which generally achieved the lowest $R^2_Y$ (see Supplementary Table S3 for the wheat dataset and Supplementary Table S4 for the maize datasets).

## Discussion

### Accounting for G$\times$E in genomic prediction

In METs, G$\times$E can be seen as resulting from QTL showing specific allele effects depending on the environmental conditions. This specificity of QTL allele effects results in genetic values that are also specific to environmental conditions. The use of a multivariate mixed model with an unstructured genetic covariance matrix between trials (here $\varvec{\Omega }_G$) is a flexible approach to capture G$\times$E in genomic prediction. The modeling of trial-specific genetic variances enables to account for differences in genetic variability between trials. It may result from the convergence/divergence of genetic values according to environmental gradients (Malosetti et al. 2013). In addition, modeling the genetic correlations between trials can take into account differences in genotype ranking between trials. This phenomenon is commonly referred to as crossovers in the G$\times$E literature (Malosetti et al. 2013). Note that the advantage of an unstructured modeling of $\varvec{\Omega }_G$ is illustrated here for a small number of trials (from $T=2$ to $T=5$) compared to what can be observed in large METs. The main drawback of this modeling is the difficulty associated with estimating parameters correctly when the number of trials becomes large (Burgueño et al. 2012). For larger datasets, factor analysis approaches can help to structure the covariance matrix $\varvec{\Omega }_G$ and reduce the number of parameters to estimate (Smith et al. 2001; de los Campos and Gianola , 2007). Compared to MGBLUP, applying a GBLUP model implies either (i) considering a common genetic variance for all trials and a genetic correlation $\rho _{t,t'}=1$ between trials when applied to the whole design (here SGBLUP), or (ii) considering a specific genetic variance in each trial but with a genetic correlation $\rho _{t,t'}=0$ between trials when applied separately in each trial (here WGBLUP).

In our study, accounting for G$\times$E using MGBLUP was more beneficial for the prediction of trial-specific performances compared to the mean performance over trials. When the set of trials represents the targeted environment, the mean performance is the variable of interest for a breeder. However, on a broader scale, breeding programs are divided into agroclimatic zones (AGZs). In this context, it is possible to apply genomic prediction jointly to all AGZs and focus on the prediction specific to each AGZ. For environments with low heritability, this joint modeling helps to improve the accuracy of genomic prediction using the information from correlated environments, as shown here based on simulated traits (see the comparison between WGBLUP and MGBLUP for trial-1 and $\rho _{t,t'}=0.8$ in Fig. 2 and Fig. S1). For the wheat dataset, despite a significant genetic correlation between drought and heat trials, no such gains could be observed for DTH and DTM which could be explained by the relatively high plot heritabilities obtained for both traits in both trials (Supplementary Table S5). The absence of gains observed for both maize datasets probably resulted from the low genetic correlations between all trial pairs (Supplementary Table S6). However, as shown with simulations and real traits, MGBLUP always enabled the highest (or close to the highest) $R^2_G$, and is thus our recommended model to apply genomic prediction for a wide range of G$\times$E scenarios.

### Forecasting the reliability of genomic prediction using CD

When applying genomic prediction in METs with G$\times$E, the precision with which each genetic value is predicted may be highly variable depending on the genotype. For instance, if one aims to predict the genetic value of a genotype in a given trial, the precision will not only depend on the number of observations for this genotype in the targeted trial and correlated trials, but also on the number of observations for other related genotypes in those trials. In unbalanced experimental designs with complex pedigree relationships between genotypes, this heterogeneity in $R^2_G$ can be very high. A breeder can be interested in assessing the $R^2_G$ associated with each genotype as it will have an impact on the selection (e.g., a genotype associated with a low $R^2_G$ is not likely to be selected using truncation selection).

As MGBLUP is a multivariate linear mixed model, it is possible to derive CD criteria that can be used to forecast and assess the $R^2_G$ prior to any validation. Our $CD_{it}$ criterion quantifies the expected $R^2_G$ associated with the prediction of a genetic value in a given trial. It consists of the adaptation of the criterion originally presented by Wientjes et al. (2015) for genomic prediction in structured populations, or Ben-Sadoun et al. (2020) for multi-trait genomic prediction, to the context of genomic prediction in METs with G$\times$E. In addition, we propose a new indicator ($CD_{i.}$) that quantifies the expected $R^2_G$ associated with the prediction of the mean genetic value of a genotype over a set of trials. When genetic values are correlated between trials, the precision with which the mean genetic value is predicted is superior to the mean of the precision achieved in each trial, and such a gain in precision is accounted for by $CD_{i.}$ (see Table 3 for an illustration).

In our study, we showed based on simulations that the expected $R^2_G$ of the prediction of both the trials-specific performance and the mean performance based on $CD_{it}$ and $CD_{i.}$ were good indications of the level of observed $R^2_G$. While this is true when averaged over a large number of traits, the expected $R^2_G$ based on CD criteria, may poorly forecast the $R^2_G$ for a specific trait (Rabier et al. 2016; Schopp et al. 2017; Rincent et al. 2017a; Rio et al. 2019). This is because CD only accounts for genetic and error variances that are not sufficient to reflect the variability in trait genetic architectures (e.g., number, genome location, and effect size of QTL). An additional source of error consists of the differences between the true genomic relationship matrix based on QTL and the one estimated based on SNPs, as previously pointed by Goddard et al. (2011). In our simulations, where an infinitesimal model was assumed, the variability of observed $R^2_G$ for a given level of expected $R^2_G$ (see Fig. 3 and Supplementary Fig. S2) could also be explained by (i) the uncertainty on $\varvec{\Omega }_G$ and $\varvec{\Omega }_E$ estimates, (ii) the uncertainty on the correlation estimate due to limited sample size, and (iii) the lack of independence between the observations used to compute the correlation due to relatedness. The latter is of high importance as it tells us that CD criteria may poorly forecast the observed $R^2_G$ when complex pedigree relationships are found in the TS. An extreme example would be a TS of genotypes that all have a high CD but are strongly related to each other so that it is difficult to predict which of them performs the best. An alternative consists of using generalized CD criteria associated with contrasts between genetic values (Laloë 1993; Rincent et al. 2012, 2017a).

### Optimizing multi-environment trials experimental designs using CD

While the cost of genotyping is becoming cheaper and should continue to decrease over the next decades, the cost of phenotyping is likely to remain expensive. These costs translate into constraints on the number of plots to which genotypes can be allocated. It is therefore important to select the genotypes whose evaluation will allow accurate prediction of the entire breeding germplasm available.

In genomic prediction, phenotypic observations for a genotype can help to predict the genetic value of other genotypes to which it is related. In this context, it is often more favorable not to replicate genotypes within and across trials, but to observe as many genotypes as possible in the overall design, as illustrated here (see Fig. 4 and Supplementary Fig. S4) and as previously shown by Endelman et al. (2014), Moehring et al. (2014), González-Barrios et al. (2019), Jarquin et al. (2020). A common related statement is that one should aim at replicating alleles rather than genotypes so that all QTL effects can be well estimated to accurately predict genetic values (Lorenz 2013). Interestingly, the interest of limiting replicates can be predicted using our new CD criteria averaged over TS genotypes (see Fig. 4 and Supplementary Fig. S4). These criteria can even be used to rank designs according to their expected $R^2_G$, which on average was shown to be consistent with their ranking according to the observed $R^2_G$.

Thanks to this consistency, optimization criteria based on $CD_{it}$ or $CD_{i.}$ can be used to optimize experimental designs that allow for higher $R^2_G$ compared to standard designs where genotypes are selected randomly from the set of candidates. The most difficult parts of this optimization procedure consist of (i) the choice of an efficient optimization algorithm and (ii) the choice of parameters $\varvec{\Omega }_G$ and $\varvec{\Omega }_E$ to be used in CD-based criteria. For the optimization algorithm, we have opted for an algorithm implemented in the R-package "TrainSel" that is a combination of GA and SAA algorithms that allows one to efficiently prospect the set of solutions while preventing local optimums. Regarding the choice of parameters, our simulation results show that experimental designs obtained from the contrasted genetic configuration S, W, and M were similar in terms of $R^2_G$, regardless of the level of genetic correlation between trials. It suggests that the choice of trait parameters has a relatively low impact on the optimization procedure, as previously shown for univariate optimizations based on CD with the impact of the heritability parameter (Rincent et al. 2012). However, the S configuration generally led to the lowest $R^2_G$ for simulated traits, and to a larger extent the lowest $R^2_Y$ for real traits. Assuming perfectly correlated genetic values between trials may thus be a too simplistic hypothesis when G$\times$E is expected in the experiment, and supports the use of new CD criteria (with configuration W or M depending on the importance of G$\times$E). While the CD is associated with the mean performance ($CD_{i.}$) is valuable to assess the $R^2_G$ for an experiment, designs optimized based on this criterion with the M configuration did not allow for improvement in $R^2_G$ regarding the mean performance (Supplementary Table S2, S3 and S4). We thus recommend using $CD_{it}$ as an optimization criterion as it is less computationally demanding.

In this study, we chose a conservative approach in that we computed the optimization criterion on all the candidates, excluding the TS used for validation. This corresponds to a scenario where we aim at checking the ability of our experimental design to predict another sample from the same population as one of the candidates. When the target population is different from the population of candidates, one can compute directly the criterion on the TS, making a targeted optimization as proposed by Akdemir and Isidro-Sánchez (2019).

## Conclusion

In this study, we demonstrated the value of a genomic prediction model that accounts for G$\times$E by modeling the genetic covariance between trials. We derived new CD criteria from this model and showed how they can help to assess and forecast the $R^2_G$ associated with the prediction of genetic values for both the trial-specific and the mean performance. We also showed how MET experimental designs could be optimized based on these new CD criteria and enabled higher $R^2_G$ compared to standard experimental designs.

From a plant breeding perspective, we propose the following guidelines when setting up and analysing a MET experiment with genomic information available: (i) analyse data from trials jointly by accounting for G$\times$E in the modeling, (ii) use indicators like CD to assess the reliability of each prediction before any validation, (iii) limit the replication of genotypes within trials and between trials (provided that genotypes are carefully allocated between trials), and (iv) allocate genotypes to trials using genomic information as well as prior information on the level of G$\times$E and heritability (e.g., using the procedure described in this study). Note that the latter guideline may be more adapted to the context of screening within a large set of individuals rather than the fine characterization of a small set of individuals for which several observations in all trials remain needed.

### Supplementary Information

Below is the link to the electronic supplementary material.

Supplementary file1 (PDF 1304 kb)

Supplementary file2 (ZIP 4 kb)

## Alternative CD derivations

An equivalent derivation of $CD_{it}$ to the one presented in Eq. (2) can be obtained following Henderson’s mixed model equations:

$$\begin{aligned} {\begin{matrix} CD_{it}=&\dfrac{\left[ \varvec{G}-\left( \varvec{Z}^T\varvec{M}_2\varvec{Z} +\varvec{G}^{-1}\right) ^{-1}\right] _{it,it}}{\varvec{G}_{it,it}}, \end{matrix}} \end{aligned}$$

where $\varvec{M}_2=\varvec{R}^{-1}-\varvec{R}^{-1}\varvec{X}\left( \varvec{X}^T\varvec{R}^{-1}\varvec{X}\right) \varvec{X}^T\varvec{R}^{-1}$. This alternative derivation can be more efficiently calculated when the dimension of $\varvec{G}$ is smaller than that of $\varvec{\Sigma }$, due to the size of matrices to be inverted.

Similarly, an equivalent derivation of $CD_{i.}$ to the one presented in Eq. (3) can be obtained:

$$\begin{aligned} {\begin{matrix} CD_{i.}=&\dfrac{\sum ^{T}_{t=1}\sum ^{T}_{t'=1}\left[ \varvec{G}-\left( \varvec{Z}^T\varvec{M}_2\varvec{Z} +\varvec{G}^{-1}\right) ^{-1}\right] _{it,it}}{\sum ^{T}_{t=1}\sum ^{T}_{t'=1}\varvec{G}_{it,it}} \end{matrix}} \end{aligned}$$

All CD formulas can also be considered using variance ratios and genetic correlations between trials where $\varvec{G}=\varvec{\Omega }_G\bigotimes \varvec{K}$ is replaced by $\widetilde{\varvec{G}}=\varvec{\Psi }\bigotimes \varvec{K}$ with $$\varvec{\Psi }=\begin{bmatrix}1 &{} .. &{}\rho _{1,T}\\ .. &{} .. &{} .. \\ \rho _{T,1} &{} .. &{} 1\end{bmatrix}$$ being the matrix of genetic correlations between trials, and $\varvec{R}=\varvec{\Omega }_E\bigotimes \varvec{I}_P$ is replaced by $\widetilde{\varvec{R}}=\varvec{\Lambda }\bigotimes \varvec{I}_P$ with $$\varvec{\Lambda }=\begin{bmatrix}\lambda _1 &{} .. &{}0\\ .. &{} .. &{} .. \\ 0 &{} .. &{} \lambda _T\end{bmatrix}$$ being the matrix of variance ratios $\lambda =\frac{\sigma ^2_{E_{t}}}{\sigma ^2_{G_{t}}}$. The latter formalism may be more convenient than using directly error and genetic covariances when one aims to forecast the $R^2_G$ or optimize the experimental design based on the level of heritability and the importance of G$\times$E.

## Footnotes

1 Available at https://github.com/QuantGen/MTM.

2 Available at https://github.com/TheRocinante-lab/TrainSel
