# Supplementary material for: Assessment of genomic prediction reliability and optimization of experimental designs in multi-environment trials
Source: Theor Appl Genet. 2021 Nov 22;135(2):405–19. doi: 10.1007/s00122-021-03972-2 (PMC8866390; doi:10.1007/s00122-021-03972-2)
Supplement: Supplementary file 1 — Supplementary file1 (PDF 1304 kb) [file 122_2021_3972_MOESM1_ESM.pdf]

Supplementary Materials

Assessment of genomic prediction reliability and optimization of experimental designs in multi-environment trials

Simon Rio[1], Deniz Akdemir[2], Tiago Carvalho[1], and Julio Isidro y Sánchez[1]

[1]Centro de Biotecnologia y Genómica de Plantas (CBGP, UPM-INIA) Universidad Politécnica de Madrid (UPM) - Instituto Nacional de Investigación y Tecnologia Agraria y Alimentaria (INIA) Campus de Montegancedo-UPM 28223-Pozuelo de Alarcón, (Madrid), Spain
[2]Agriculture & Food Science, Animal and Crop section, University College Dublin, Dublin, Ireland

September 1, 2021

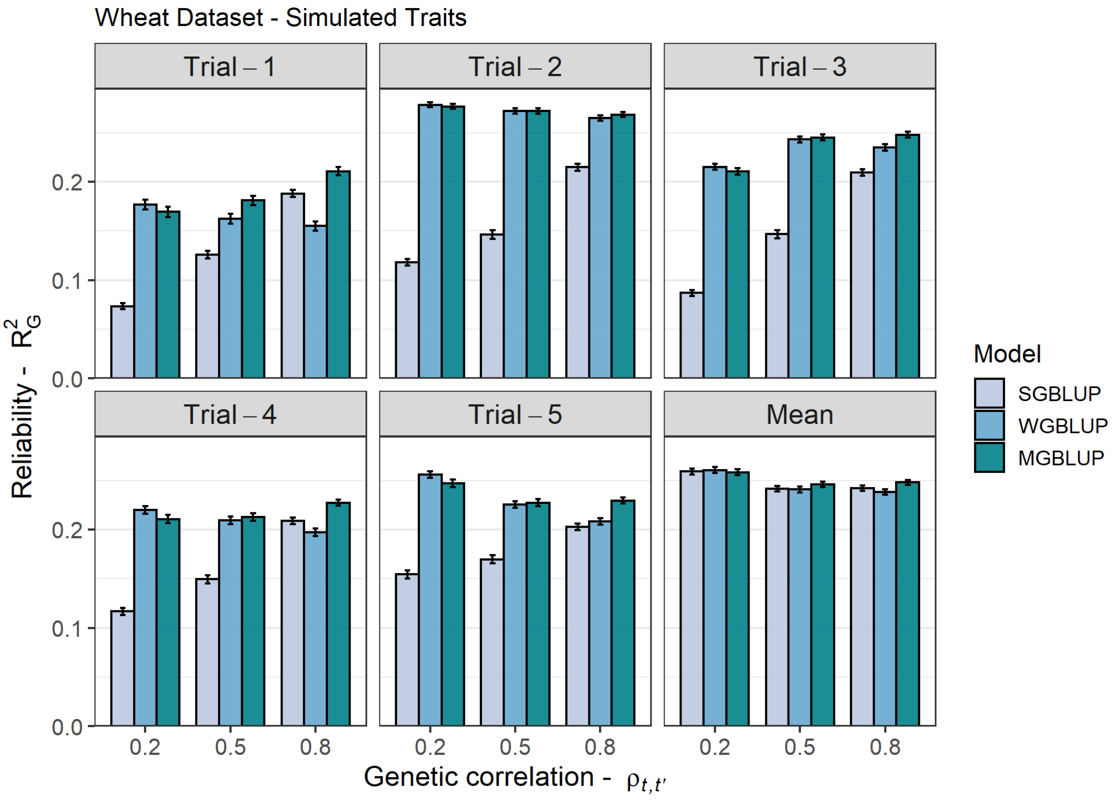

Supplementary Figure S1. Average genomic prediction reliability (R^2_G) of the trial-specific performance ($T = 5$ with different h^2_t, see Table 1) and the mean performance of the test set according to the genomic prediction model: SGBLUP, WGBLUP or MGBLUP. The average R^2_G was computed over 30 simulated traits using the wheat dataset and 30 cross-validation replicates for each level of genetic correlation $\rho_{t,t'}$, and $\pm$ the standard error of the 30 CV replicates averaged over the 30 traits is indicated by a bar.

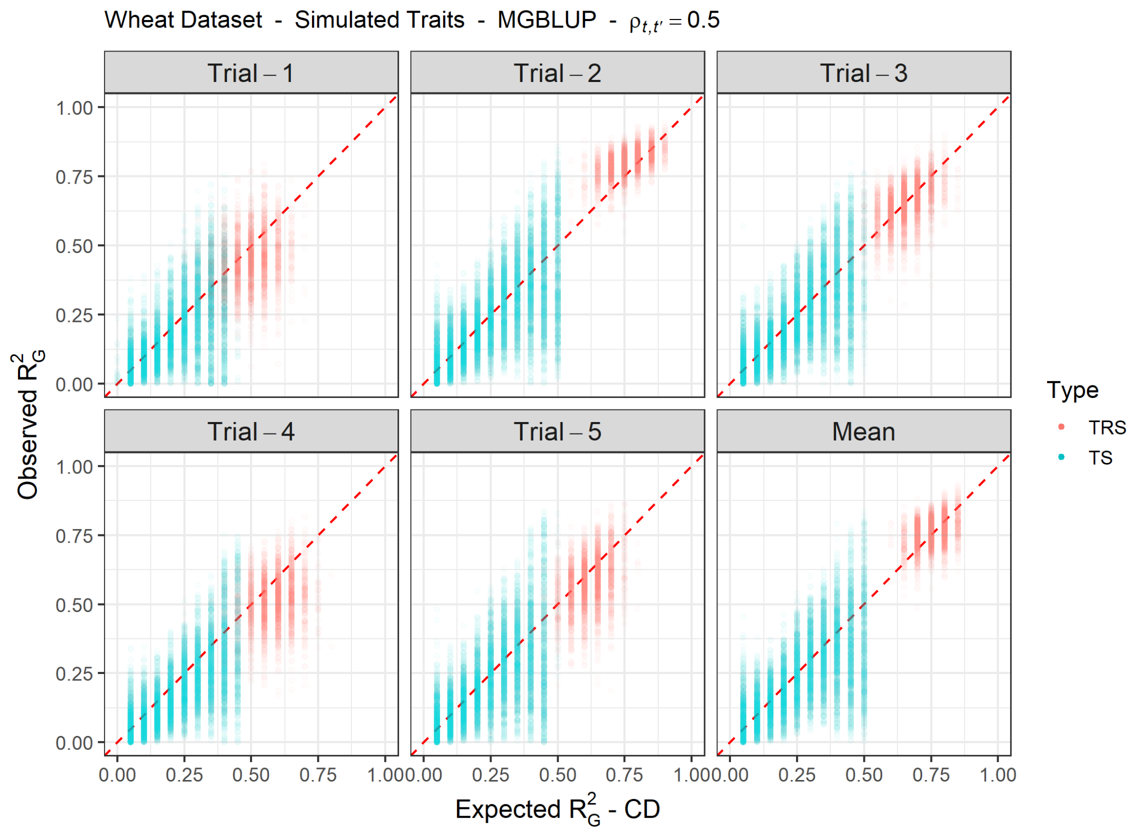

Supplementary Figure S2. Observed genomic prediction reliability (R_G^2) against expected R_G^2 for 30 simulated traits ($\rho_{t,t'} = 0.5$ and $T = 5$ with different h_t^2, see Table 1) using the wheat dataset and 30 cross-validation replicates for the training set (TRS) and the test set (TS). Expected R_G^2 is based on CD_{it} for trial-specific performances and $CD_{i.}$ for the mean performance. Individual CD values are clustered into intervals of similar CD using a sliding window approach. The observed R_G^2 of the interval is then computed based on genomic predictions obtained with MGBLUP.

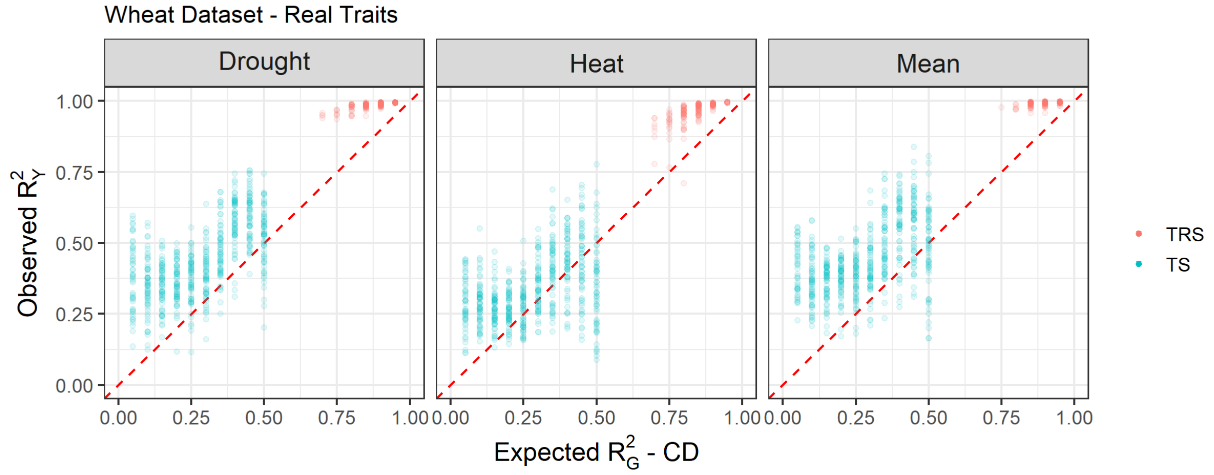

Supplementary Figure S3. Observed phenotype-based genomic prediction reliability (R^2_Y) against expected R^2_G using the wheat dataset for days-to-heading (DTH) and days-to-maturity (DTM) based on 30 cross-validation replicates. Expected R^2_G is based on CD_{it} for trial-specific performances and $CD_{i.}$ for the mean performance. Individual CD values are clustered into intervals of similar CD using a sliding window approach. The observed R^2_Y of the interval is then computed based on genomic predictions obtained with MGBLUP.

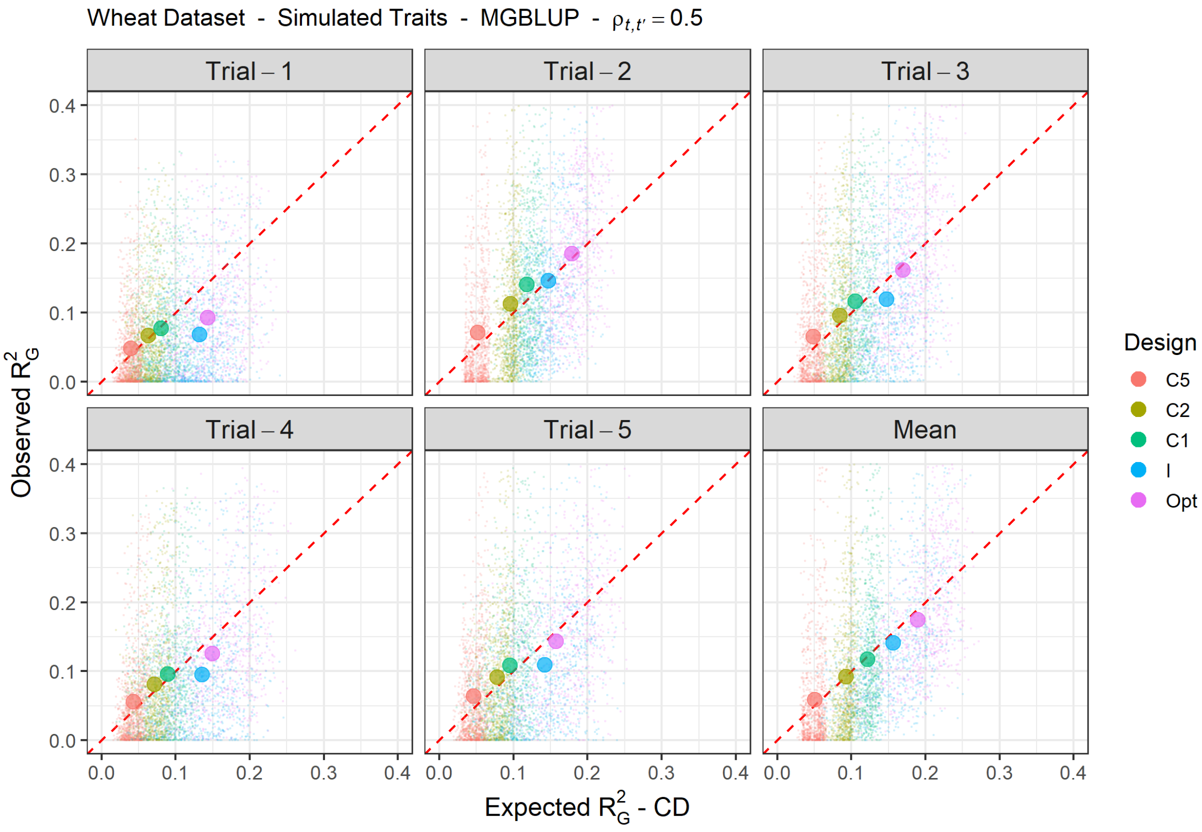

Supplementary Figure S4 Observed genomic prediction reliability (R_G^2) obtained with MGBLUP against expected R_G^2 of the test set according to the experimental design: complete with k replicates (Ck), incomplete and unreplicated (I), and optimized based on CD_{it} with the M configuration (see Table 2), for 30 simulated traits ($\rho_{t,t'} = 0.5$ and $T = 5$, with different h_t^2, see Table 1) using the wheat dataset and 30 design replicates. Expected R_G^2 is based on the mean over test set individuals of CD_{it} for trial-specific performances and $CD_{i.}$ for the mean performance. For a given type of design, the average observed R_G^2 against the average expected R_G^2 is represented by a big dot.

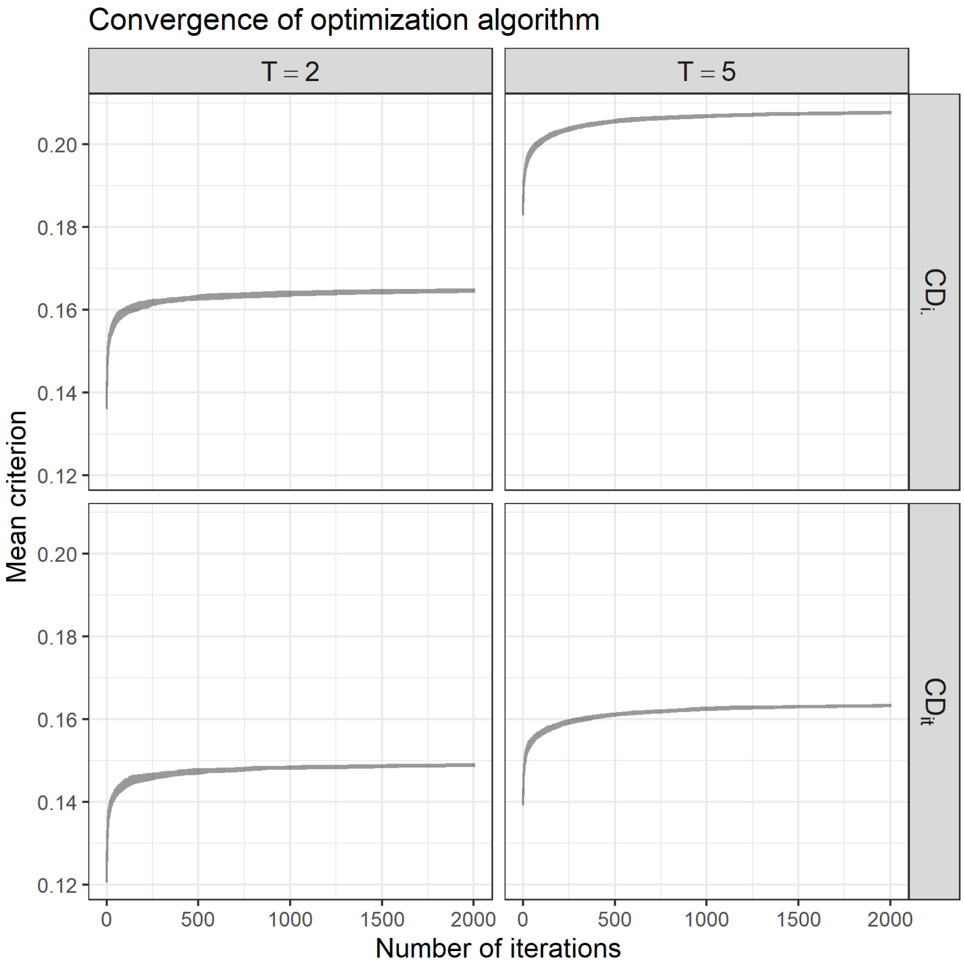

Supplementary Figure S5. Evolution of the optimization criteria (mean of CD_{it} or $CD_{i.}$) according to the number of iterations for $T = 2$ or $T = 5$ trials, based on the M optimization configuration and $P = 50$ plots per trial using the wheat dataset. All genotypes were considered as candidates here and each run only differed for the initial set of solutions.

Criterion	T	$T \times P$	N_c
Mean of CD_{it}	2	100	44.1 (3.9)
Mean of CD_{it}	5	250	121.0 (6.0)
Mean of $CD_{i.}$	2	100	42.9 (4.0)
Mean of $CD_{i.}$	5	250	109.8 (6.2)

Supplementary Table S1. Table summarizing, for different optimization procedures based on the M optimization configuration and $P = 50$ plots per trial using the wheat dataset, the optimization criterion (mean of CD_{it} or $CD_{i.}$), the number of trials (T), the total number of plots ($T \times P$), the number of common genotypes selected (N_c) between pairs of optimized designs along with the standard deviation between brackets.

T	$\rho_{t,t'}$	Conf.	Crit.	Trial-1	Trial-2	Trial-3	Trial-4	Trial-5	Mean
2	0.2	S	CD_i^S	0.07 (0.04)	0.18 (0.01)	-	-	-	0.13 (0.01)
		W	CD_{it}^W	0.08 (0.01)	0.19 (0.01)	-	-	-	0.14 (0.01)
		M	CD_{it}^M	0.08 (0.01)	0.19 (0.01)	-	-	-	0.14 (0.01)
		M	$CD_{i.}^M$	0.08 (0.01)	0.19 (0.01)	-	-	-	0.14 (0.01)
	0.5	S	CD_i^S	0.10 (0.01)	0.15 (0.01)	-	-	-	0.14 (0.01)
		W	CD_{it}^W	0.11 (0.01)	0.16 (0.01)	-	-	-	0.15 (0.01)
		M	CD_{it}^M	0.10 (0.01)	0.16 (0.01)	-	-	-	0.15 (0.01)
		M	$CD_{i.}^M$	0.10 (0.01)	0.16 (0.01)	-	-	-	0.14 (0.01)
	0.8	S	CD_i^S	0.16 (0.01)	0.21 (0.01)	-	-	-	0.21 (0.01)
		W	CD_{it}^W	0.17 (0.01)	0.22 (0.01)	-	-	-	0.22 (0.01)
		M	CD_{it}^M	0.17 (0.01)	0.22 (0.01)	-	-	-	0.22 (0.01)
		M	$CD_{i.}^M$	0.17 (0.01)	0.22 (0.01)	-	-	-	0.22 (0.01)
5	0.2	S	CD_i^S	0.07 (0.01)	0.16 (0.01)	0.10 (0.01)	0.10 (0.01)	0.14 (0.01)	0.16 (0.01)
		W	CD_{it}^W	0.09 (0.01)	0.19 (0.01)	0.12 (0.01)	0.13 (0.01)	0.16 (0.01)	0.17 (0.01)
		M	CD_{it}^M	0.09 (0.01)	0.19 (0.01)	0.11 (0.01)	0.12 (0.01)	0.15 (0.01)	0.17 (0.01)
		M	$CD_{i.}^M$	0.08 (0.01)	0.18 (0.01)	0.11 (0.01)	0.12 (0.01)	0.15 (0.01)	0.17 (0.01)
	0.5	S	CD_i^S	0.08 (0.01)	0.17 (0.01)	0.15 (0.01)	0.11 (0.01)	0.13 (0.01)	0.17 (0.01)
		W	CD_{it}^W	0.10 (0.01)	0.19 (0.01)	0.16 (0.01)	0.13 (0.01)	0.15 (0.01)	0.18 (0.01)
		M	CD_{it}^M	0.11 (0.01)	0.19 (0.01)	0.17 (0.01)	0.13 (0.01)	0.15 (0.01)	0.18 (0.01)
		M	$CD_{i.}^M$	0.09 (0.01)	0.18 (0.01)	0.16 (0.01)	0.12 (0.01)	0.14 (0.01)	0.18 (0.01)
	0.8	S	CD_i^S	0.12 (0.01)	0.19 (0.01)	0.17 (0.01)	0.15 (0.01)	0.15 (0.01)	0.19 (0.01)
		W	CD_{it}^W	0.13 (0.01)	0.20 (0.01)	0.18 (0.01)	0.16 (0.01)	0.16 (0.01)	0.19 (0.01)
		M	CD_{it}^M	0.13 (0.01)	0.21 (0.01)	0.19 (0.01)	0.16 (0.01)	0.17 (0.01)	0.20 (0.01)
		M	$CD_{i.}^M$	0.13 (0.01)	0.21 (0.01)	0.19 (0.01)	0.16 (0.01)	0.16 (0.01)	0.20 (0.01)

Supplementary Table S2. Average genomic prediction reliability (R_G^2) obtained with MGBLUP for the trial-specific ($T = 2$ or $T = 5$ with different h_t^2, see Table 1) and the mean performance of the test set according to the experimental design optimized based on new CD criteria (CD_{it} and $CD_{i.}$) using the S, W and M configurations (see Table 2). The average R_G^2 was computed over 30 simulated traits using the wheat dataset and 30 design replicates for each level of genetic correlation $\rho_{t,t'}$, and the standard error of the 30 design replicates averaged over the 30 traits is shown between brackets. Note that CD_{it}^W was indicated but not $CD_{i.}^W$ as averaging both criteria over test set individuals results to identical quantities, and thus identical optimizations.

Trait	Conf.	Crit.	Drought	Heat	Mean
	S	CD_i^S	0.252 (0.011)	0.135 (0.011)	0.223 (0.011)
DTH	W	CD_{it}^W	0.250 (0.010)	0.128 (0.008)	0.212 (0.008)
	M	CD_{it}^M	0.250 (0.010)	0.152 (0.008)	0.228 (0.009)
	M	$CD_{i.}^M$	0.254 (0.011)	0.152 (0.008)	0.235 (0.010)
	S	CD_i^S	0.209 (0.010)	0.092 (0.010)	0.155 (0.011)
DTM	W	CD_{it}^W	0.240 (0.008)	0.123 (0.009)	0.170 (0.009)
	M	CD_{it}^M	0.224 (0.010)	0.152 (0.011)	0.189 (0.008)
	M	$CD_{i.}^M$	0.229 (0.009)	0.135 (0.013)	0.187 (0.010)

Supplementary Table S3. Average phenotype-based genomic prediction reliability (R_Y^2) obtained with MGBLUP using the wheat dataset for days-to-heading (DTH) and days-to-maturity (DTM) regarding the trial-specific performance (drought and heat) and the mean performance of the test set according to the experimental design optimized based on new CD criteria (CD_{it} and $CD_{i.}$) using the S, W and M configurations (see Table 2). The average R_Y^2 was computed over 30 design replicates and the standard error is shown between brackets. Note that CD_{it}^W was indicated but not $CD_{i.}^W$ as averaging both criteria over test set individuals results to identical quantities, and thus identical optimizations.

Trait	Conf.	Crit.	Drought	Kiboko	Kmega	Mean
	S	CD_i^S	0.078 (0.008)	0.056 (0.007)	0.265 (0.014)	0.181 (0.012)
T1	W	CD_{it}^W	0.095 (0.009)	0.078 (0.008)	0.242 (0.013)	0.176 (0.011)
	M	CD_{it}^M	0.079 (0.009)	0.080 (0.008)	0.264 (0.014)	0.201 (0.010)
	M	$CD_{i.}^M$	0.090 (0.009)	0.062 (0.008)	0.256 (0.015)	0.171 (0.010)
	S	CD_i^S	0.309 (0.020)	0.410 (0.019)	0.375 (0.016)	0.550 (0.014)
T2	W	CD_{it}^W	0.340 (0.017)	0.416 (0.021)	0.378 (0.016)	0.541 (0.013)
	M	CD_{it}^M	0.344 (0.019)	0.420 (0.022)	0.403 (0.012)	0.556 (0.013)
	M	$CD_{i.}^M$	0.328 (0.020)	0.407 (0.020)	0.373 (0.016)	0.545 (0.010)

Supplementary Table S4. Average phenotype-based genomic prediction reliability (R_Y^2) obtained with MGBLUP using the maize datasets T1 and T2 for grain yield regarding the trial-specific performance (Drought, Kiboko and Kmega) and the mean performance of the test set according to the experimental design optimized based on new CD criteria (CD_{it} and $CD_{i.}$) using the S, W and M configurations (see Table 2). The average R_Y^2 was computed over 30 design replicates and the standard error is shown between brackets. Note that CD_{it}^W was indicated but not $CD_{i.}^W$ as averaging both criteria over test set individuals results to identical quantities, and thus identical optimizations.

	DTH	DTM
$\sigma^2_{G_D}$	38.99	37.51
$\sigma^2_{G_H}$	18.64	15.51
$\rho_{D,H}$	0.60	0.45
$\sigma^2_{E_D}$	4.17	13.68
$\sigma^2_{E_H}$	7.39	5.98
h^2_D	0.90	0.73
h^2_H	0.72	0.72

Supplementary Table S5. Parameters estimated using MGBLUP for days-to-heading (DTH) and days-to-maturity (DTM) in a drought (D) and heat (H) trials using all available data. Parameters include: trial-specific genetic variances ($\sigma^2_{G_t}$), error variances ($\sigma^2_{E_t}$), plot heritabilities (h^2_t), and genetic correlations between trials ($\rho_{t,t'}$).

	T1	T2
$\sigma^2_{G_D}$	0.168	0.162
$\sigma^2_{G_{Ki}}$	0.448	0.515
$\sigma^2_{G_{Km}}$	0.972	0.797
$\rho_{D,Ki}$	0.20	0.34
$\rho_{D,Km}$	0.20	-0.09
$\rho_{Ki,Km}$	0.38	0.32
$\sigma^2_{E_D}$	0.318	0.343
$\sigma^2_{E_{Ki}}$	0.878	0.574
$\sigma^2_{E_{Km}}$	1.458	1.499
h^2_D	0.35	0.32
h^2_{Ki}	0.34	0.47
h^2_{Km}	0.40	0.35

Supplementary Table S6. Parameters estimated using MGBLUP for grain yield (t/ha) using maize populations T1 and T2 evaluated in three trials (D: Drought, Ki: Kibiko and Km: Kmega) using all available data. Parameters include: trial-specific genetic variances ($\sigma^2_{G_t}$), error variances ($\sigma^2_{E_t}$), plot heritabilities (h^2_t), and genetic correlations between trials ($\rho_{t,t'}$).
